# Triglyceride–glucose index as a marker of adverse cardiovascular prognosis in patients with coronary heart disease and hypertension

**DOI:** 10.1186/s12933-023-01866-9

**Published:** 2023-06-09

**Authors:** Yahui Liu, Binbin Zhu, Weicen Zhou, Yao Du, Datun Qi, Chenxu Wang, Qianqian Cheng, You Zhang, Shan Wang, Chuanyu Gao

**Affiliations:** 1 Department of Cardiology, Zhengzhou University People’s Hospital, Henan Provincial People’s Hospital, Zhengzhou, China; 2 Henan Provincial Key Lab for Control of Coronary Heart Disease, Zhengzhou University Central China Fuwai Hospital, Zhengzhou, 451464 China

**Keywords:** Triglyceride–glucose index, Coronary heart disease, Hypertension, Adverse cardiovascular events

## Abstract

**Background:**

The triglyceride–glucose (TyG) index has been proposed as a potential predictor of adverse prognosis of cardiovascular diseases (CVDs). However, its prognostic value in patients with coronary heart disease (CHD) and hypertension remains unclear.

**Methods:**

A total of 1467 hospitalized patients with CHD and hypertension from January 2021 to December 2021 were included in this prospective and observational clinical study. The TyG index was calculated as Ln [fasting triglyceride level (mg/dL) × fasting plasma glucose level (mg/dL)/2]. Patients were divided into tertiles according to TyG index values. The primary endpoint was a compound endpoint, defined as the first occurrence of all-cause mortality or total nonfatal CVDs events within one-year follow up. The secondary endpoint was atherosclerotic CVD (ASCVD) events, including non-fatal stroke/transient ischemic attack (TIA) and recurrent CHD events. We used restricted cubic spline analysis and multivariate adjusted Cox proportional hazard models to investigate the associations of the TyG index with primary endpoint events.

**Results:**

During the one-year follow-up period, 154 (10.5%) primary endpoint events were recorded, including 129 (8.8%) ASCVD events. After adjusting for confounding variables, for per standard deviation (SD) increase in the TyG index, the risk of incident primary endpoint events increased by 28% [hazard ratio (HR) = 1.28, 95% confidence interval (CI) 1.04–1.59]. Compared with subjects in the lowest tertile (T1), the fully adjusted HR for primary endpoint events was 1.43 (95% CI 0.90–2.26) in the middle (T2) and 1.73 (95% CI 1.06–2.82) in highest tertile (T3) (*P* for trend = 0.018). Similar results were observed in ASCVD events. Restricted cubic spline analysis also showed that the cumulative risk of primary endpoint events increased as TyG index increased.

**Conclusions:**

The elevated TyG index was a potential marker of adverse prognosis in patients with CHD and hypertension.

**Supplementary Information:**

The online version contains supplementary material available at 10.1186/s12933-023-01866-9.

## Introduction

With the rapid development of the global economy and the increasingly serious aging, about 31.1% of adults in the world are affected by hypertension [1]. Especially in China, the prevalence of hypertension surged from 5.1% in 1958 to 27.9% in 2015 [2]. As a major risk factor for atherosclerosis, hypertension often coexists with CHD. The incidence and mortality of CHD are on the rise and are becoming a serious public health problem worldwide [3].

More and more evidence show that insulin resistance (IR) and its related diseases are not only a sign of diabetic patients, but also a risk factor for CVDs in non-diabetic patients. In recent years, TyG index has been favored by many researchers because it is a very easy to obtain value to evaluate the degree of IR [4]. The results of many studies have shown that the increase of TyG index is not only related to the increase of the incidence of CVDs, such as hypertension [5, 6], atherosclerotic cardiovascular disease (ASCVD) [7–9], metabolic related diseases [10, 11], but also can predict the adverse consequences of patients with hypertension or CHD [12, 13]. However, there is still a lack of research on whether TyG can predict the poor prognosis of patients with CHD and hypertension. The purpose of this study was to investigate whether TyG index has predictive value for the occurrence of major adverse events in patients with CHD and hypertension, and further to explore the correlation between TyG index and cardiovascular events in different subgroups.

## Methods

### Study design and participants

This trial was a single-center, prospective observational, hospital-based clinical trial approved by the Ethics Committee of Zhengzhou University Central China Fuwai Hospital (Fuwai Central China Cardiovascular Hospital) (Approval No. 2021–20). This study was in accordance with the principles of the Declaration of Helsinki and all patients completed the signing of informed consent form before being enrolled.

**Fig. 1:**
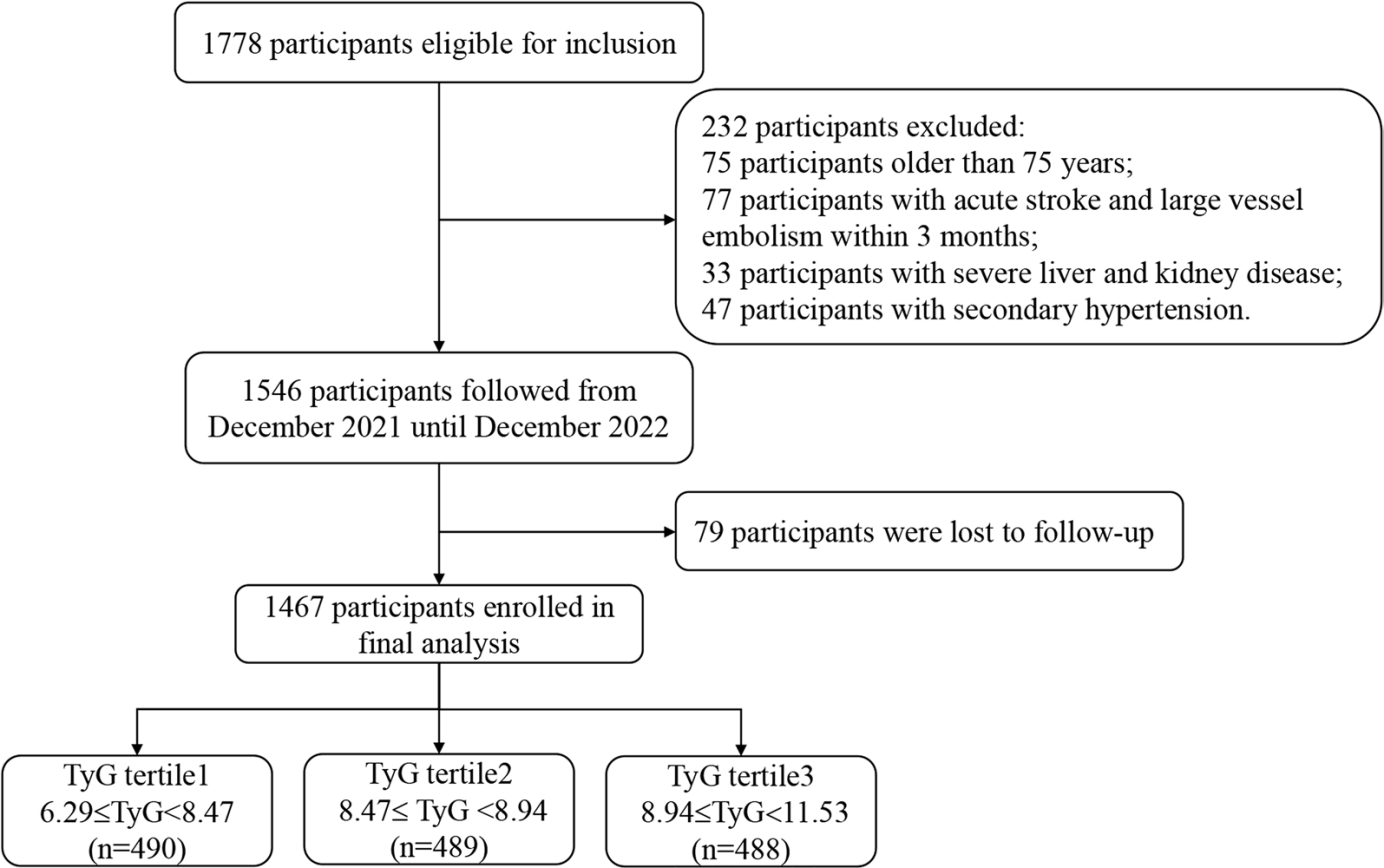
Flow diagram of patient selection

From January 2021 to December 2021, a total of 1778 subjects aged 18 to 75 years old with CHD and hypertension who had a previous history or were first diagnosed in the inpatient department were enrolled in accordance with the trial procedure (Fig. 1). Patients who met the following conditions were excluded: (1) patients younger than 18 years or older than 75 years; (2) patients diagnosed with secondary hypertension; (3) pregnant patients; (4) patients with history of acute stroke, pulmonary embolism and large vessel embolism within 3 months; (5) patients with severe mental disorders, combined with severe respiratory failure, malignant tumor disease, liver and kidney disease, and other life expectancy less than 1 year were excluded.

### Data collection and definitions

Following enrollment, baseline data on each patient was collected, including personal information, the clinical history, laboratory indicators, and medical imaging data. Personal information included weight, height [to calculate body mass index (BMI)], baseline blood pressure, age, gender, smoking history and drinking history; the clinical history included established history of CVDs [including myocardial infarction (MI) or heart failure (HF) or stroke)], diabetes, chronic kidney disease (CKD), peripheral artery disease (PAD); the laboratory indicators of blood samples were fasting venous blood collected by professional medical staff from all participants in the early morning, including fasting blood glucose (FBG), glycated hemoglobin A1c (HbA1c), total cholesterol (TC), triglyceride (TG), high density lipoprotein cholesterol (HDL-C), low density lipoprotein cholesterol (LDL-C), estimated glomerular filtration rate (eGFR) and uric acid (UA); imaging medical data included the location and extent of coronary artery disease. The units of FBG and TG were first converted from mmol/L to mg/dL, and the calculation basis was multiplied by 18.02 and 88.54, respectively. Then, the TyG index was calculated as Ln [fasting TG (mg/dL) × FBG (mg/dL)/2].

CHD was defined as having at least one of the following conditions [14]: (1) percutaneous coronary angiography or computed tomographic angiography (CTA) examination showed that at least one coronary artery trunk or primary branch had ≥ 50% stenosis; (2) typical exertional angina symptoms with positive stress test (electrocardiogram stress test, stress echocardiography or nuclide myocardial stress imaging); (3) previously diagnosed MI; (4) previously diagnosed unstable angina pectoris (typical ischemic chest pain + ECG changes + increased markers of muscle damage; or the dynamic changes of ST segment during ischemic attack, or coronary angiography confirmed the existence of severe lesions leading to symptoms).

The WHO hypertension guidelines were used to define the initial diagnosis of hypertension defined as systolic blood pressure (SBP) was greater than 140 mmHg or their diastolic blood pressure (DBP) was no less than 90 mmHg [15]. All patients were considered to two groups according to blood pressure control: controlled hypertension (blood pressure < 140/90 mmHg on three or less antihypertensive drugs); uncontrolled hypertension (blood pressure ≥ 140/90 mmHg on unlimited types of antihypertensive drugs or < 140/90 mmHg on at least four or more antihypertensive drugs including diuretic).

### Follow-Up and endpoints

Clinical follow-up was carried out by skilled clinicians in outpatient or telephone contact at the time points of 1 month, 6 months, and 12 months, and standard computerized case report forms were filled out at each of these intervals. When there was more than one endpoint event occurred during the follow-up period, the first event data were statistically analyzed. The endpoint events were independently categorized by three cardiovascular specialists who were not aware of the baseline information. When there were disagreements regarding event identification, the three experts came to a decision together after talking.

The primary endpoint of this clinical trial was defined as a compound endpoint of all-cause mortality or the first occurrence of total nonfatal CVDs events within one-year follow up. The total CVDs was defined as follows: (1) nonfatal stroke/TIA, defined as the sudden onset of a neurological deficit persisting for ≥ 24 h in the absence of any other disease that could account for the symptoms, with the findings of brain computed tomography or magnetic resonance imaging; (2) nonfatal CHD, defined as acute MI, unstable angina requiring coronary revascularization [percutaneous coronary intervention (PCI) or coronary artery bypass grafting (CABG)]; (3) HF, defined as requiring hospitalization and treatment due to clinical manifestations of HF. The secondary endpoint was ASCVD events, defined as the occurrence of stroke/TIA and CHD events.

### Statistical analysis

All statistical analyses were performed using SPSS version 27.0 (SPSS, Chicago, IL) and R version 4.2.3. The subjects were classified according to the occurrence of primary endpoint events during follow-up and the tertiles of TyG index. Continuous data that met the normality test was expressed as mean ± SD or median (interquartile range), and categorical variables were expressed as percentages (%). The continuous variables included age, BMI, TC, TG, HDL-C, LDL-C, FBG, HbA1c, eGFR and UA; categorical variables included gender, smoking, drinking, past medical history and usage of medications. One-way analysis of variance (for continuous data) or Fisher’s exact test or χ^2^ test (for categorical data) was used to compare the demographic variables and clinical characteristics and the results were Bonferroni’s corrected (for multiple comparisons). The cumulative incidence of endpoint events in groups were analyzed using the Kaplan–Meier curve.

In order to evaluate whether the TyG index affects the occurrence of endpoint events, we constructed three Cox proportional hazard regression models. The selection of confounding factors was based on statistically significant (*P* < 0.05) variables with primary endpoint events in univariate Cox analysis and clinically important variables: Cox Model 1 was unadjusted model; Model 2 was adjusted by confounding factors including age, sex; Model 3 was further adjusted for current smoking, BMI, diabetes, established CVDs (including MI, stroke, HF), stains, antiplatelet drugs, fibrates drugs, antidiabetic agents, SBP, DBP, UA, eGFR, TC, LDL-C. The TyG index was input into the model as a continuous variable and a categorical variable (the tertile of the TyG index). The dose–response relationship between the TyG index and the risk of the primary endpoint events was analyzed by performing the restricted cubic spline analysis. We also performed subgroup analysis based on gender, age (< 65 years or ≥ 65 years), BMI (< 28.0 kg/m^2^ or ≥ 28.0 kg/m^2^), diabetes status (yes or no), established CVDs (yes or no), blood pressure control status (controlled or uncontrolled) and LDL-C level (< 1.80 mmol/L or ≥ 1.80 mmol/L) to determine whether the correlation between TyG index and primary endpoint events in different subgroups was different, and the *P* value of the interaction was calculated. *P* value of less than 0.05 was defined as statistically significant.

## Results

### Baseline characteristics of the patient population

**Fig. 2:**
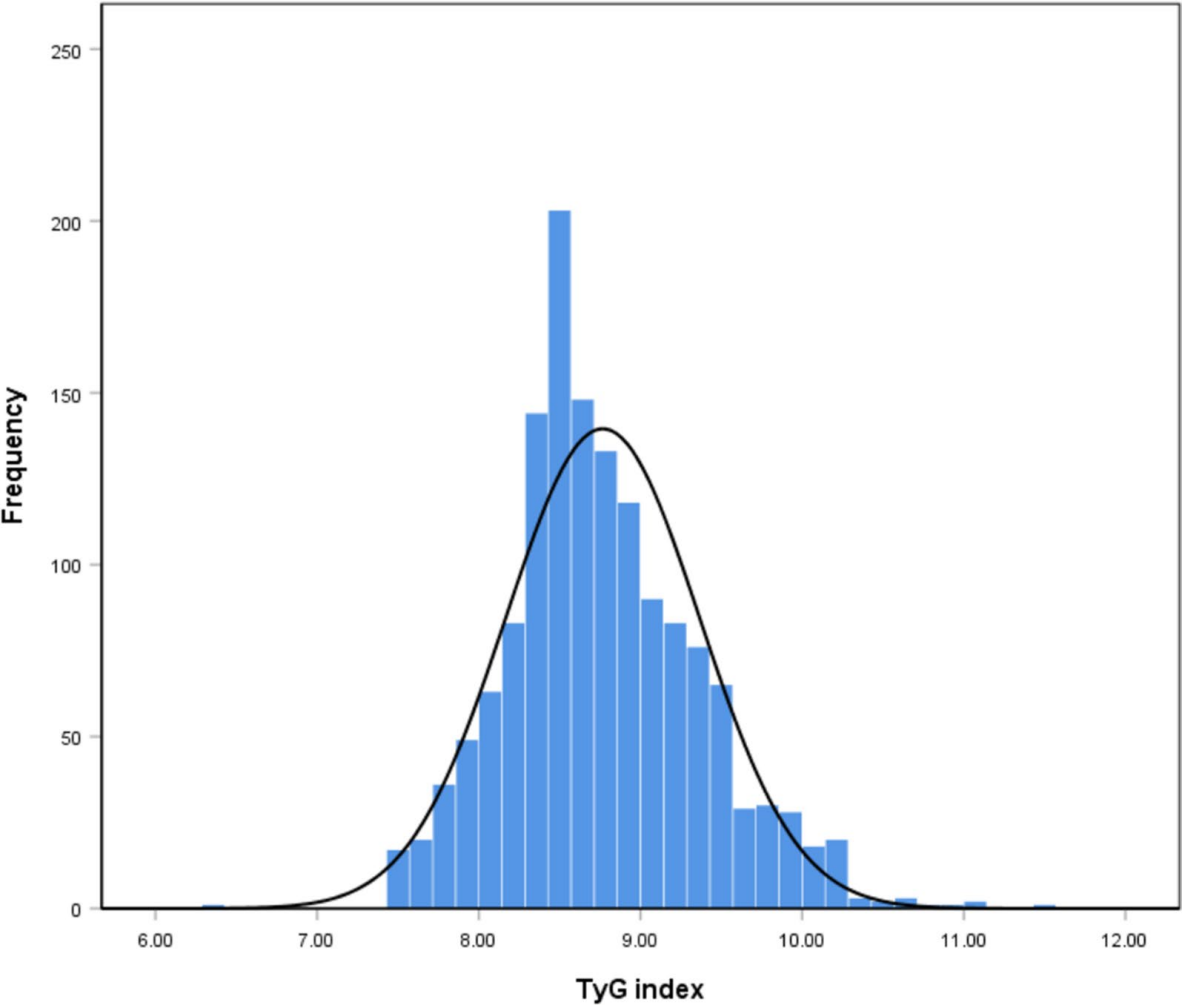
Distribution of the TyG index in all patients

A total of 1467 patients with CHD and hypertension were enrolled in this study, with an average age of 60.5 ± 9.4 years, and 1021 (69.6%) patients were male. All patients were grouped according to the tertile level of TyG index (tertile 1: 6.29 ≤ TyG < 8.47; tertile 2: 8.47 ≤ TyG < 8.94; tertile 3: 8.94 ≤ TyG ≤ 11.53) (Fig. 1). The distribution of TyG index in the population was shown in Fig. 2. The baseline clinical characteristics of all the patients by tertiles of TyG were presented in Table 1. The proportions of individuals with diabetes, PAD, fat, usage of β-Blocker and antidiabetic agents were significantly higher in the highest TyG index tertile, as well as the level of BMI, FBG, HbA1c, TG, TC, LDL-C. Meanwhile, the highest TyG index tertile had younger age and lower HDL-C (all *P* < 0.05). However, there was no significant difference in gender distribution, CVDs and CKD prevalence, target vessel territory, usage of stains and antiplatelet medication, SBP and eGFR level among the three groups (all *P* > 0.05, Table 1).

**Table 1:** Baseline characteristics of total and groups by TyG index tertile

Variables	TyG index tertile				
	Total (n = 1467)	6.29 ≤ T1 < 8.47 (n = 490)	8.47 ≤ T2 < 8.94 (n = 489)	8.94 ≤ T3 ≤ 11.53 (n = 488)	*P* value*
Age (years)	60.5 ± 9.4	61.4 ± 9.1	61.0 ± 9.3	59.1 ± 9.5	** < 0.001**
Male (n, %)	1021 (69.6)	359 (73.3)	324 (66.3)	338 (69.3)	0.057
BMI (kg/m^2^)	26.8 ± 3.3	26.5 ± 3.4	26.7 ± 3.1	27.1 ± 3.2	**0.005**
BMI ≥ 28.0	456 (31.1)	135 (27.6)	145 (29.7)	176 (36.1)	**0.011**
SBP (mmHg)	134.1 ± 15.2	133.5 ± 15.0	133.3 ± 15.3	135.3 ± 15.4	0.083
DBP (mmHg)	82.3 ± 10.1	82.3 ± 9.9	81.2 ± 10.1	83.5 ± 10.2	**0.002**
Current smoking (n, %)	360 (24.5)	109 (22.2)	112 (22.9)	139 (28.5)	**0.045**
Current drinking (n, %)	262 (17.9)	72 (14.7)	92 (18.8)	98 (20.1)	0.071
Case history (n, %)					
CVDs	497 (33.9)	162 (33.9)	166 (33.1)	169 (34.6)	0.884
Diabetes	685 (46.7)	135 (27.6)	213 (43.6)	337 (69.1)	** < 0.001**
PAD	290 (19.8)	78 (15.9)	85 (17.4)	128 (26.0)	** < 0.001**
CKD^a^	124 (8.5)	32 (6.5)	43 (8.8)	49 (10.0)	0.135
Number of coronary lesions (n, %)					
One‑vessel disease	362 (24.7)	137 (28.0)	113 (23.1)	112 (23.0)	0.357
Two‑vessel disease	345 (23.5)	111 (22.7)	119 (24.3)	115 (23.6)	
Three‑vessel disease	760 (51.8)	242 (49.4)	257 (52.6)	262 (53.5)	
Target vessel territory (n, %)					
LM	110 (7.5)	36 (7.3)	38 (7.8)	36 (7.4)	0.961
LAD	1035 (70.6)	345 (70.4)	339 (69.3)	351 (71.9)	0.669
LCX	820 (55.9)	272 (55.5)	273 (55.8)	275 (56.4)	0.965
RCA	828 (56.4)	274 (55.9)	270 (55.2)	284 (58.2)	0.617
Cardiovascular medications (n, %)					
ACEI/ARB	1049 (71.5)	336 (68.6)	353 (72.2)	360 (73.8)	0.182
β-Blocker	1140 (77.7)	362 (73.9)	388 (79.3)	390 (79.9)	**0.043**
Antiplatelet medication	1444 (98.4)	482 (98.4)	483 (98.8)	479 (98.2)	0.732
Stains	1420 (96.9)	473 (96.9)	476 (97.3)	471 (96.5)	0.756
Fibrate	4 (0.3)	0	0	4 (0.8)	**0.018**
Antidiabetic agents	621 (42.4)	124 (25.4)	187 (38.2)	310 (63.5)	** < 0.001**
Metformin	317 (21.6)	58 (11.8)	87 (17.8)	172 (35.2)	** < 0.001**
Alpha-glucosidase inhibitor	277 (18.9)	54 (11.0)	71 (14.5)	152 (31.1)	** < 0.001**
Sulfonylurea	78 (5.3)	20 (4.1)	20 (4.1)	38 (7.8)	**0.012**
SGLT2i	309 (21.1)	53 (10.8)	90 (18.4)	166 (34.0)	** < 0.001**
Dipeptidyl peptidase-4 inhibitor	27 (1.8)	5 (1.0)	9 (1.8)	13 (2.7)	0.161
Insulin	131 (8.9)	22 (4.5)	33 (6.7)	76 (15.6)	** < 0.001**
GLP-1 receptor agonist	30 (2.0)	1 (0.2)	4 (0.8)	25 (5.1)	** < 0.001**
Laboratory variables					
TC (mmol/L)	3.47 (2.99–4.18)	3.15 (2.76–3.58)	3.54 (3.04–4.20)	3.91 (3.23–4.53)	** < 0.001**
TG (mmol/L)	1.36 (1.02–1.84)	0.95 (0.78–1.11)	1.39 (1.19–1.60)	2.15 (1.66–2.81)	** < 0.001**
HDL-C (mmol/L)	1.36 (1.02–1.84)	1.06 (0.94–1.25)	1.01 (0.88–1.17)	0.94 (0.79–1.07)	** < 0.001**
LDL-C (mmol/L)	1.00 (0.87–1.15)	1.70 (1.37–2.08)	1.96 (1.54–2.54)	2.13 (1.61–2.69)	** < 0.001**
FBG (mmol/L)	5.40 (4.70–6.46)	4.78 (4.31–5.38)	5.38 (4.78–6.04)	6.69 (5.54–8.73)	** < 0.001**
HbA1c (%)	5.93 (5.42–6.95)	5.55 (5.21–6.12)	5.86 (5.43–6.64)	6.78 (5.82–7.98)	** < 0.001**
UA (umol/L)	309.0 (256.0–374.0)	300.0 (247.0–366.0)	307.0 (253.5–368.0)	323.0 (265.0–400.0)	** < 0.001**
eGFR (mL/min/1.73 m^2^)	86.8 ± 17.1	86.8 ± 15.5	86.7 ± 16.7	87.0 ± 18.9	0.959

TyG index, triglyceride–glucose index; BMI, body mass index; SBP, systolic blood pressure; DBP, diastolic blood pressure; CVDs, cardiovascular diseases; PAD, peripheral artery disease; CKD, chronic kidney disease; ACEI, angiotensin-converting enzyme inhibitors; ARB, angiotensin II receptor blockers; SGLT2i, sodium-glucose cotransporter 2 inhibitor; GLP-1, Glucagon-like peptide-1; TC, total cholesterol; HDL-C, high density lipoprotein cholesterol; LDL-C, low density lipoprotein cholesterol; FBG, fasting blood glucose; HbA1c, glycated hemoglobin A1c; UA, uric acid; eGFR, estimated glomerular filtration rate

^a^Defined as eGFR < 60 ml/min/1.73 m^2^ on the basis of The KDIGO CKD Clinical Guideline. Statistical significance was defined as *P* < 0.05. *P* values in bold are < 0.05

^*^For multiple comparisons

### Baseline characteristics of the group stratified by the occurrence of primary endpoint events

Baseline characteristics of participants with and without primary endpoint events were shown in Table 2. Patients in whom with primary endpoint events developed tended to have more CVDs (*P* = 0.021) and CKD prevalence (*P* < 0.001), or to have multivessel disease (*P* = 0.020) and LCX vessel territory (*P* = 0.006). Significant differences could also be found for TC level (*P* = 0.030), UA level (*P* = 0.020), eGFR level (*P* < 0.001), usage of metformin (*P* = 0.005) and sulfonylurea agents (*P* = 0.003).

**Table 2:** Baseline characteristics of patients with and without primary endpoint events

Variables	Without events (n = 1313)	With events (n = 154)	*P* value
Age (years)	60.4 ± 9.3	61.5 ± 9.7	0.160
Male (n, %)	907 (69.1)	114 (74.0)	0.229
BMI (kg/m^2^)	26.8 ± 3.3	26.8 ± 3.1	0.913
BMI ≥ 28.0	403 (30.7)	53 (34.4)	0.345
SBP (mmHg)	133.9 ± 15.1	135.7 ± 16.0	0.155
DBP (mmHg)	82.3 ± 10.0	83.0 ± 11.2	0.416
Current smoking (n, %)	321 (24.4)	39 (25.3)	0.811
Current drinking (n, %)	235 (17.9)	27 (17.5)	0.911
Case history (n, %)			
CVDs	432 (32.9)	65 (42.2)	**0.021**
Diabetes	603 (45.9)	82 (53.2)	0.085
PAD	252 (19.2)	38 (24.7)	0.106
CKD^a^	98 (7.5)	26 (16.9)	** < 0.001**
Number of coronary lesions (n, %)			**0.020**
One‑vessel disease	337 (25.7)	25 (16.3)	
Two‑vessel disease	310 (23.6)	35 (22.7)	
Three‑vessel disease	666 (50.7)	94 (61.0)	
Target vessel territory (n, %)			
LM	93 (7.1)	17 (11.0)	0.078
LAD	920 (70.1)	115 (74.7)	0.235
LCX	718 (54.7)	102 (66.2)	**0.006**
RCA	732 (55.8)	96 (62.3)	0.119
Cardiovascular medications (n, %)			
ACEI/ARB	935 (71.2)	114 (74.0)	0.464
β-Blocker	1013 (77.2)	127 (82.5)	0.134
Antiplatelet medication	1292 (98.4)	152 (98.7)	0.776
Stains	1270 (96.9)	150 (97.4)	0.718
Fibrates	4 (0.3)	0	0.493
Antidiabetic agents	548 (41.8)	73 (47.4)	0.181
Metformin	270 (20.6)	47 (30.5)	**0.005**
Alpha-glucosidase inhibitor	248 (18.9)	29(18.8)	0.986
Sulfonylurea	62 (4.7)	16 (10.4)	**0.003**
SGLT2i	270 (20.6)	39 (25.3)	0.170
Dipeptidyl peptidase-4 inhibitor	23 (1.8)	4 (2.6)	0.460
Insulin	120 (9.1)	11 (7.1)	0.411
GLP-1 receptor agonist	27 (2.1)	3 (1.9)	0.928
Laboratory variables			
TC (mmol/L)	3.47 (3.00–4.17)	3.50 (2.91–4.23)	0.482
TG (mmol/L)	1.33 (1.02–1.84)	1.45 (1.08–1.94)	**0.030**
HDL-C (mmol/L)	1.00 (0.87–1.15)	1.02 (0.87–1.14)	0.762
LDL-C (mmol/L)	1.91 (1.50–2.47)	1.93 (1.48–2.36)	0.488
FBG (mmol/L)	5.39 (4.70–6.44)	5.59 (4.87–6.69)	0.116
HbA1c (%)	5.91 (5.41–6.94)	6.02 (5.49–7.03)	0.166
UA (umol/L)	308.0 (254.5–371.0)	326.0 (262.5–417.5)	**0.020**
eGFR (mL/min/1.73 m^2^)	87.5 ± 16.7	81.5 ± 19.4	** < 0.001**

Data were given as mean ± SD, median with interquartile range or n (%)

BMI, body mass index; SBP, systolic blood pressure; DBP, diastolic blood pressure; CVDs, cardiovascular diseases; PAD, peripheral artery disease; CKD, chronic kidney disease; ACEI, angiotensin-converting enzyme inhibitors; ARB, angiotensin II receptor blockers; SGLT2i, sodium-glucose cotransporter 2 inhibitor; GLP-1, Glucagon-like peptide-1; TC, total cholesterol; HDL-C, high density lipoprotein cholesterol; LDL-C, low density lipoprotein cholesterol; FBG, fasting blood glucose; HbA1c, glycated hemoglobin A1c; UA, uric acid; eGFR, estimated glomerular filtration rate.

^a^Defined as eGFR < 60 ml/min/1.73 m^2^ on the basis of The KDIGO CKD Clinical Guideline. *P* values in bold are < 0.05

### Correlations between the TyG index and cardiovascular risk factors.

We used Spearman or Pearson correlation analysis to test the correlation between TyG index and cardiovascular risk factors. The results showed that TyG index was positively correlated with BMI, DBP, TC, TG, LDL-C and UA and negative correlated with age, HDL-C (all *P* < 0.05). There was no significant correlation between TyG index and SBP and eGFR (all *P* > 0.05) (Table 3).

**Table 3:** The correlation between TyG index and baseline clinical risk factors

Variables	Correlation coefficient (r)	*P* value
Age (years)	− 0.159^a^	< 0.001
BMI (kg/m^2^)	0.131^a^	< 0.001
SBP (mmHg)	0.048^a^	0.068
DBP (mmHg)	0.060^a^	0.022
TC (mmol/L)	0.366^b^	< 0.001
TG (mmol/L)	0.864^b^	< 0.001
HDL-C (mmol/L)	− 0.303^b^	< 0.001
LDL-C (mmol/L)	0.250^b^	< 0.001
FBG (mmol/L)	0.577^b^	< 0.001
HbA1c (%)	0.434^b^	< 0.001
UA (umol/L)	0.128^b^	< 0.001
eGFR (mL/min/1.73 m^2^)	0.023^a^	0.387

BMI, body mass index; SBP, systolic blood pressure; DBP, diastolic blood pressure; TC, total cholesterol; TG, triglyceride; HDL-C, high density lipoprotein cholesterol; LDL-C, low density lipoprotein cholesterol; FBG, fasting blood glucose; HbA1c, glycated hemoglobin A1c; UA, uric acid; eGFR, estimated glomerular filtration rate

^a^Pearson correlation analysis

^b^Spearman correlation analysis

### Relationship between the TyG index and cardiovascular events

**Fig. 3:**
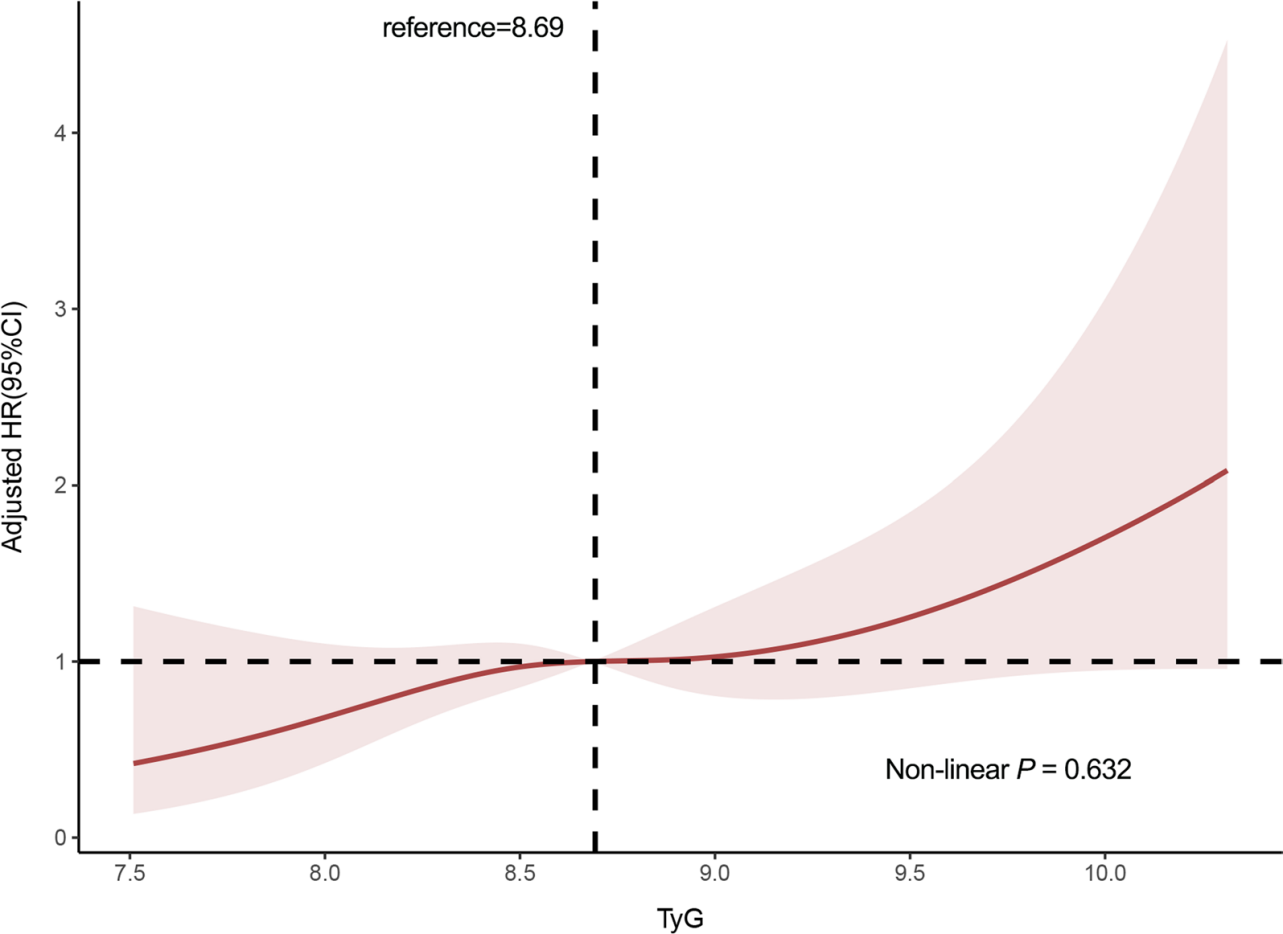
Multivariable-adjusted HR for primary endpoint events based on restricted cubic spines for the TyG index. Red lines represented references for HR, and red areas represent 95% CI. HR was adjusted for age, gender, current smoking, BMI, diabetes, established CVDs (including MI, stroke, HF), stains, antiplatelet drugs, fibrates drugs, antidiabetic agents, SBP, DBP, UA, eGFR, TC, LDL-C in the multivariate model. HR, hazard ratio; CI, confidence interval; BMI, body mass index; MI, myocardial infarction; HF, heart failure; SBP, systolic blood pressure; DBP, diastolic blood pressure; UA, uric acid; eGFR, estimated glomerular filtration rate; TC, total cholesterol; LDL-C, low-density lipoprotein cholesterol

**Fig. 4:**
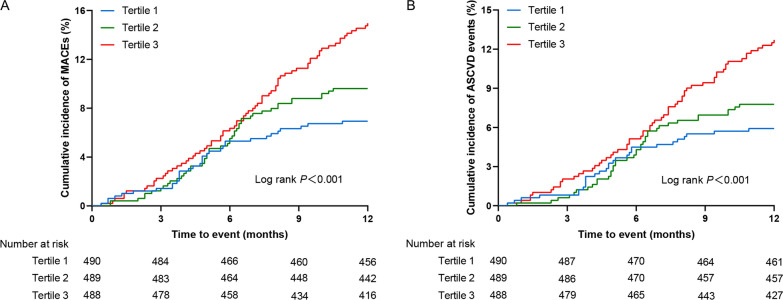
Kaplan–Meier curves for endpoint events according to tertile of TyG index. **A** Kaplan–Meier curves for primary endpoint events; **B** Kaplan–Meier curves for ASCVD events

The results of the restricted cubic splines were presented in Fig. 3. We observed a dose–response relationship between the TyG index and risk of primary endpoint events (non-linear *P* = 0.632). All patients were followed up for one year after discharge. During the follow-up period, 154 (10.5%) primary endpoint events were recorded, including 129 (8.8%) ASCVD events. To show the outcomes of patients with different levels of the TyG index, we generated unadjusted Kaplan–Meier survival plots (Fig. 4, Additional file 2: Fig. S1). As shown in Fig. 4, the cumulative incidence of both primary endpoint events and ASCVD events increased incrementally across tertiles of the TyG index (all Log rank *P* < 0.001).

Cox regression analyses were implemented to explore the independent risk factors for primary endpoint events and ASCVD events. The screening of confounding factors in the multivariate Cox proportional hazards model was mainly based on statistically significant clinical variables after univariate Cox regression analyses and the results showed five statistically significant variables (established CVDs, CKD, UA, eGFR, TyG) (Additional file 1: Table S1). Then, we also incorporated confounding variables (age, gender, current smoking, BMI, diabetes, stains, antiplatelet drugs, fibrates, antidiabetic agents, SBP, DBP, TC, LDL-C) that may affect the clinical prognosis of CHD into the model 3 to obtain more accurate HR results as much as possible. Multivariate Cox proportional hazards regression analysis showed that the TyG index, whether considered as a categorical or continuous variable, remained significant after adjusting for confounders. For per SD increase in the TyG index, the risk of incident primary endpoint events increased by 41% (HR = 1.41; 95% CI 1.23–1.67) in the partially adjusted regression model 2. Compared with subjects in the lowest tertile (T1), the partially adjusted HR for primary endpoint events was 1.45 (95% CI 0.93–2.26) in the middle (T2) and 2.38 (95% CI 1.58–3.59) in the highest tertile (T3), respectively. The increased risk of primary endpoint events from T1 to T3 was statistically significant (*P* for trend < 0.001). A similar pattern was observed in fully adjusted model (Per SD increase: HR = 1.28, 95% CI 1.04–1.59; Tertile 2: HR = 1.43, 95% CI 0.90–2.26; Tertile 3: HR = 1.73, 95% CI 1.06–2.82; *P* for trend = 0.018) (Table 4).

**Table 4:** HR (95% CI) of outcomes according to TyG index in the three Models

Outcomes	n	Model 1^a^	Model 2^b^	Model 3^c^
		HR (95% CI)	HR (95% CI)	HR (95% CI)
TyG index as a continuous variable				
Per 1 unit increase				
Primary endpoint events	154 (10.5)	1.67 (1.31–2.13)^***^	1.82 (1.42–2.35)^**^	1.52 (1.07–2.17)^*^
ASCVD events	129 (8.8)	1.72 (1.32–2.24)^**^	1.83 (1.38–2.39)^**^	1.55 (1.06–2.26)^*^
Per 1 SD increase				
Primary endpoint events	154 (10.5)	1.36 (1.18–1.57)^**^	1.41 (1.23–1.67)^**^	1.28 (1.04–1.59)^*^
ASCVD events	129 (8.8)	1.39 (1.18–1.62)^**^	1.43 (1.22–1.69)^**^	1.30 (1.04–1.63)^*^
TyG index as a nominal variable				
Primary endpoint events	154 (10.5)			
T1	34 (6.9)	1 [Reference]	1 [Reference]	1 [Reference]
T2	47 (9.6)	1.40 (0.90–2.17)	1.45 (0.93–2.26)	1.43 (0.90–2.26)
T3	73 (15.0)	2.21 (1.47–3.32)^***^	2.38 (1.58–3.59)^**^	1.73 (1.06–2.82)^*^
P for trend		< 0.001	< 0.001	0.018
ASCVD events	129 (8.8)			
T1	29 (5.9)	1 [Reference]	1 [Reference]	1 [Reference]
T2	38 (7.8)	1.32 (0.81–2.14)	1.31 (0.81–2.13)	1.37 (0.83–2.25)
T3	62 (12.7)	2.19 (1.41–3.41)^***^	2.29 (1.47–3.58)^**^	1.77 (1.05–3.00)^*^
P for trend		0.001	0.001	0.012

Model 1 was unadjusted model. Model 2 was adjusted for age, gender. Model 3 was adjusted for variables in model 2 plus current smoking, BMI, diabetes, established CVDs (including MI, stroke, HF), stains, antiplatelet drugs, fibrates drugs, antidiabetic agents, SBP, DBP, UA, eGFR, TC, LDL-C in the multivariate model. HR, hazard ratio; CI, confidence interval; BMI, body mass index; MI, myocardial infarction; HF, heart failure; SBP, systolic blood pressure; DBP, diastolic blood pressure; UA, uric acid; eGFR, estimated glomerular filtration rate; TC, total cholesterol; LDL-C, low-density lipoprotein cholesterol

^*^P < 0.05

^**^P < 0.01

^***^P < 0.001

Moreover, we further studied the associations between the TyG index and ASCVD events. For per SD increase in the TyG index, the risk of incident ASCVD increased by 30% (HR = 1.30; 95% CI 1.04–1.63) in the fully adjusted regression model. Compared with subjects in the lowest tertile (T1), the fully adjusted HR for ASCVD was 1.37 (95% CI 0.83–2.25) in the middle (T2) and 1.77 (95% CI 1.05–3.00) in the highest tertile (T3), respectively (Table 4). The increased risk of ASCVD events from tertile 1 to tertile 3 was also statistically significant (*P* for trend = 0.012). Moreover, the sensitivity analysis that excluded patients with a history of antidiabetic agents and SGLT2i agent usage showed results were consistent with the primary analysis (Additional file 1: Table S2).

### Subgroup analysis

**Fig. 5:**
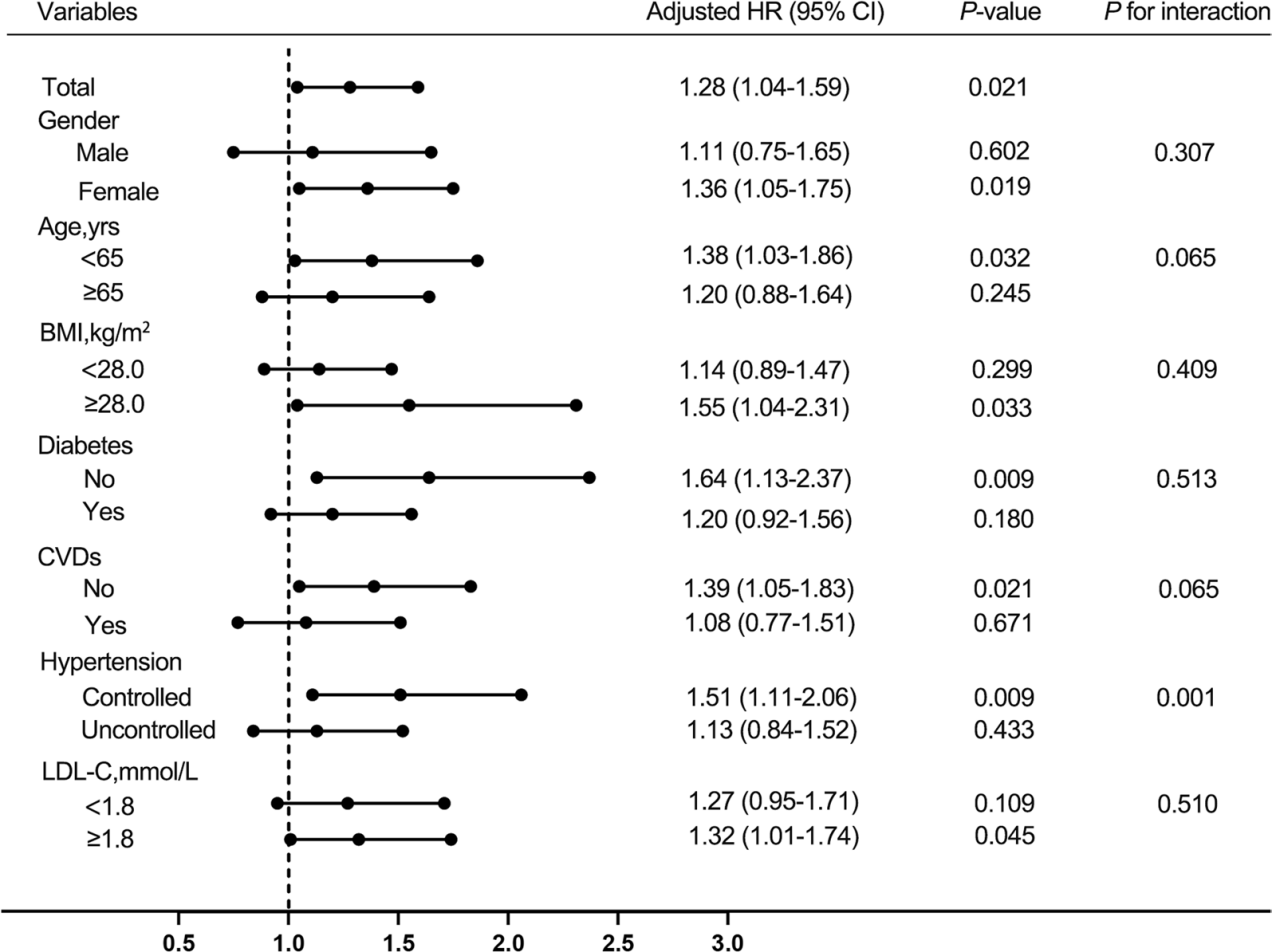
Cox proportional hazards analysis evaluating prognostic implication of TyG index in various subgroups. HR was evaluated by 1‑SD increase of TyG index. HR was adjusted for age, gender, current smoking, BMI, diabetes, established CVDs (including MI, stroke, HF), stains, antiplatelet drugs, fibrates drugs, antidiabetic agents, SBP, DBP, UA, eGFR, TC, LDL-C in the multivariate model. The above repeated confounding factors would be removed when analyzing different subgroups. HR, hazard ratio; CI, confidence interval; BMI, body mass index; MI, myocardial infarction; HF, heart failure; SBP, systolic blood pressure; DBP, diastolic blood pressure; UA, uric acid; eGFR, estimated glomerular filtration rate; TC, total cholesterol; LDL-C, low-density lipoprotein cholesterol

The association between the TyG index and primary endpoint events was examined in the subgroups analysis according to gender (male or female), age (> 65 or ≤ 65 years), BMI (≥ 28.0 or < 28.0 kg/m^2^), diabetes states (yes or no), CVDs (yes or no), blood pressure control status (controlled or uncontrolled) and LDL-C (≥ 1.80 or < 1.80 mmol/L). There was no statistically significant interaction between gender, age, BMI, diabetes states, CVDs, LDL-C level and TyG index (all *P*-values for interaction ≥ 0.05). The statistical significance was observed only among females, patients without diabetes, patients without established CVDs, patients with BMI ≥ 28.0 kg/m^2^ and patients with LDL-C ≥ 1.80 mmol/L (all *P* < 0.05) (Fig. 5).

Interestingly, TyG level was a more significant factor in controlled hypertension than in uncontrolled hypertension [HR 1.51 (95% CI 1.11–2.06) in controlled hypertension and HR 1.13 (95% CI 0.84–1.52) in uncontrolled hypertension, *P* for interaction = 0.001]. Therefore, we further analyzed the association between the TyG index and outcomes using new multivariate Cox regression analysis with hypertension control status as a categorical confounding variable. The results showed that TyG was still a favorable predictor of adverse prognosis in patients with CHD and hypertension (Additional file 1: Table S3).

## Discussion

Recently, a large number of studies have provided strong statistical evidence for the correlation between TyG index and the development and prognosis of CVDs [4]. However, the prognostic value of TyG index in patients with CHD and hypertension remains undetermined. The results of our prospective observational study included the following three findings: (1) The TyG index, independent of traditional cardiovascular risk factors, was associated with increased risk for adverse events in patients with CHD and hypertension. (2) The TyG index was a more significant predictor for worse prognosis in patients with CHD and controlled hypertension than in those with uncontrolled hypertension. (3) The significant association between the TyG index and primary endpoint events was mainly observed among females, patients without diabetes or established CVDs, patients with BMI ≥ 28.0 kg/m^2^ or LDL-C ≥ 1.80 mmol/L.

In addition to diabetes, IR is also an important marker of metabolic syndrome such as obesity, hypertension and dyslipidemia, which have been fully proved to be closely related to CVDs [4, 16, 17]. Previous studies revealed the relationships between the TyG index and obesity, dyslipidemia and renal insufficiency [18, 19]. In the present study, our results also revealed the correlations between the TyG index and traditional cardiovascular risk factors, such as age, BMI, blood pressure, UA, blood lipid and blood glucose metabolism level. Of note, there was a negative relationship between age and TyG index in this study. Theoretically, aging can be associated with poor glucose tolerance because insulin secretion decreases with age [20]. However, previous data suggested that the inflammation that accompanies excess adiposity states, such as diabetes and IR, could be even more relevant for CHD occurring at younger ages [21]. This may explain the phenomenon that patients with CHD with higher TyG index were younger in our study and previous studies [19, 22–24].

As a convenient and easy-to-obtain measure of IR, TyG index can not only predict the occurrence of diabetes [10], hypertension [5, 25], ASCVD [1, 7, 9] and even tumor-related diseases [26, 27], but also can be used as a clinical prognostic indicator of some CVDs [4]. Laura et al. first proposed a significant correlation between TyG index and CVDs events (AUC: 0.708, 95% CI 0.68–0.73) [28]. Since then, most studies have shown that TyG index has good potential in predicting poor cardiovascular prognosis in patients with acute coronary syndrome (ACS) [29, 30], stable CHD [31, 32], non-obstructive CHD [33] or chronic total occlusion lesions[34], HF [35], atherosclerosis [36] and other CVDs [4]. Hypertension and diabetes are the most important risk factors for CVDs and play a crucial role in the occurrence and development of CHD. This means that patients with CHD with hypertension or diabetes have a worse cardiovascular prognosis, which has been confirmed in previous studies [37]. Furthermore, there is accumulating evidence suggesting that elevated TyG index is associated with adverse outcomes in CHD patients [4]. However, the TyG index for the prognostic value for patients with CHD and hypertension remains poorly known. The association between TyG index and CHD complicated with hypertension only exists in subgroup analysis of hypertension or non-hypertension in patients with ACS and chronic total occlusion lesions, and the conclusions were inconsistent or not representative due to the small sample size [34, 38, 39]. Our study shows for the first time that TyG index can be used as a prognostic indicator for CHD and hypertension, especially in patients with well controlled hypertension, which is our novel and interesting finding worthy of future research to prove and explore.

TyG level was a more significant factor in controlled hypertension than in uncontrolled hypertension. The possible explanation for this inconsistency is considered as follows: Firstly, previous data have shown that about 50% of hypertensive patients are considered to have IR and hyperinsulinemia [40]. IR and hyperinsulinemia maybe more significant in patients with CHD and controlled hypertension than in uncontrolled hypertension in this study, however, which requires further specific-designed studies to determine whether interventions of IR assessed by TyG index have a positive impact on improving clinical prognosis in this population. Secondly, uncontrolled hypertension means poor blood pressure control or diagnosis of resistant hypertension during the one-year follow-up period. For patients with uncontrolled hypertension who do not take enough antihypertensive drugs or resistant hypertension with complex pathogenesis, the effect of persistent high blood pressure status itself can lead to more severe adverse cardiovascular prognosis, which may weaken or interfere with the judgment of the true prognostic value of the TyG index in these patients [37]. Thirdly, the results of our study may have some limitations due to the limited sample size, the relatively short follow-up time and lack of dynamic monitoring the TyG index. In general, previous studies have shown that the interaction between hypertension and IR can lead to the progression of atherosclerosis [31], which means that patients with hypertension should pay close attention to IR indicators. In particular, for patients with CHD and hypertension who have more risk factors, while controlling blood pressure well, we should also pay close attention to TyG indicators to reduce or mitigate the occurrence of long-term poor prognosis.

Gender differences in IR-related CVDs risk have previously been reported [19, 41]. In our subgroup analysis, TyG index can be used as a prognostic indicator for female patients with CHD and hypertension, which may be due to the influence of gender differences in anthropometric measurements, preferred location of fat storage, heavier risk factor burden and higher TG [19, 42]. TyG index also can be used as a predictor for obese patients and patients with LDLC ≥ 1.8 mmol/L, because most of these patients have indications for IR. Otherwise, this study observed a significant correlation between TyG index and cardiovascular events in patients without diabetes, which is consistent with previous study results [19, 43], suggesting that patients with diabetes are receiving hypoglycemic therapy or developing healthier habits, so that their TyG index may be well controlled [28].

The mechanism of TyG index related to CVDs and adverse cardiovascular outcomes has not been clearly elucidated. TyG is an index composed of two risk factors for CVD, both lipid-related and glucose-related factors reflect IR in the human body, which may be one of the explanations for this association [4, 16, 44]. First of all, the imbalance of glucose metabolism and lipid metabolism caused by IR may induce inflammation and oxidative stress, and lead to the occurrence of atherosclerosis [45]. Secondly, abnormal secretion of NO associated with IR can damage the vascular endothelium and lead to endothelium-dependent vasodilation [46]. In addition, IR can also induce excessive production of reactive oxidative stress (ROS), leading to impaired endothelial function [47]. Thirdly, IR may lead to platelet overactivity and increase platelet adhesion induction and thromboxane A2 (TxA2)-dependent tissue factor expression, eventually leading to thrombosis and inflammation [48]. Finally, IR with hyperglycemia will induce excessive glycosylation, thereby promoting smooth muscle cell proliferation, collagen cross-linking and collagen deposition. The above process will lead to increased diastolic left ventricular stiffness, cardiac fibrosis, and ultimately lead to heart failure [17].

Several limitations of this trial should be considered. Firstly, this was a single-center study, so potential bias may be introduced. Secondly, the sample size was relatively small, and the incidence of all-cause death was relatively low, which may limit reliable statistical analysis and make it difficult to specify the association between the TyG index and a single component of primary endpoint events. Thirdly, insulin levels were not measured in most patients in this study, and HOMA-IR values could not be calculated. Fourthly, laboratory parameters were only detected once on admission, and changes during the one-year follow-up period may cause deviations in the analysis results. Finally, although most of the patients with CHD enrolled in the study were diagnosed by coronary angiography, some were still diagnosed by coronary CTA. Further multicenter, more rigorous studies with larger sample sizes may make our conclusions more reliable.

## Conclusions

In conclusion, the TyG index was significantly associated with adverse prognosis in patients with CHD and hypertension, and this relationship remained significant after adjustment for other confounders. Thus, in clinical work, for patients with CHD and hypertension, TyG index should be paid attention and closely monitored while strengthening the control of traditional cardiovascular risk factors including hypertension.

## Supplementary Information

**Additional file 1:** **Table S1.** Univariate Cox proportional hazard analysis between variables and primary endpoint. **Table S2.** Sensitivity analysis for the association between the TyG index and outcomes. **Table S3.** Multivariable Cox regression analysis for the association between the TyG index and outcomes.

**Additional file 2: Figure S1.** Kaplan–Meier curves for endpoint events according to quartiles of TyG index. A: Kaplan–Meier curves for primary endpoint events; B: Kaplan–Meier curves for ASCVD events.

## Data Availability

The date that supports the findings of this study are available from the corresponding author upon reasonable request. The date is not publicly due to them containing information that could compromise research participant privacy.

## Abbreviations

TyG: Triglyceride–glucose index
CVDs: Cardiovascular diseases
CHD: Coronary heart disease
ASCVD: Atherosclerotic cardiovascular disease
TIA: Transient ischemic attack
SD: Standard deviation
HR: Hazard ratio
CI: Confidence interval
IR: Insulin resistance
BMI: Body mass index
MI: Myocardial infarction
HF: Heart failure
CKD: Chronic kidney disease
PAD: Peripheral artery disease
FBG: Fasting blood glucose
HbA1c: Glycated hemoglobin A1c
TC: Total cholesterol
TG: Triglyceride
HDL-C: High density lipoprotein cholesterol
LDL-C: Low density lipoprotein cholesterol
eGFR: Estimated glomerular filtration rate
UA: Uric acid
eGFR: Estimated glomerular filtration rate
CTA: Computed tomographic angiography
SBP: Systolic blood pressure
DBP: Diastolic blood pressure
PCI: Percutaneous coronary intervention
CABG: Coronary artery bypass grafting
SGLT2i: Sodium-glucose cotransporter 2 inhibitor
AUC: Area under curve
ASC: Acute coronary syndrome
ROS: Reactive oxidative stress
