# Supplementary material for: Triglyceride–glucose index as a marker of adverse cardiovascular prognosis in patients with coronary heart disease and hypertension
Source: Cardiovasc Diabetol. 2023 Jun 9;22:133. doi: 10.1186/s12933-023-01866-9 (PMC10257289; doi:10.1186/s12933-023-01866-9)
Supplement: Supplementary file 1 — Additional file 1: Table S1. Univariate Cox proportional hazard analysis between variables and primary endpoint. Table S2. Sensitivity analysis for the association between the TyG index and outcomes. Table S3. Multivariable Cox regression analysis for the association between the TyG index and outcomes. [file 12933_2023_1866_MOESM1_ESM.docx]

**Additional file 1**

**Table S1. Univariate Cox proportional hazard analysis between variables and primary endpoint.**

| **Variables** | **HR (95% CI)** | **P value** |  |
| --- | --- | --- | --- |
| Age (years) | 1.013 (0.996-1.031) | 0.141 | |
| Gender, female | 0.792 (0.553-1.136) | 0.205 | |
| BMI (kg/m^2^) | 1.002 (0.955-1.002) | 0.925 | |
| SBP (mmHg) | 1.007 (0.997-1.018) | 0.155 | |
| DBP (mmHg) | 1.006 (0.991-1.022) | 0.441 | |
| Current smoking | 1.047 (0.728-1.506) | 0.803 | |
| Current drinking | 0.976 (0.644-1.479) | 0.910 | |
| Established CVDs | 1.464 (1.063-2.016) | **0.019** | |
| Diabetes | 1.309 (0.954-1.796) | 0.096 | |
| PAD | 1.358 (0.942-1.960) | 0.101 | |
| CKD^a^ | 2.413 (1.583-3.679) | **<0.001** | |
| LM lesion | 1.581 (0.955-2.617) | 0.075 | |
| LAD lesion | 1.240 (0.863-1.783) | 0.245 | |
| LCX lesion | 1.580 (0.937-2.207) | 0.072 | |
| RCA lesion | 1.290 (0.931-1.787) | 0.126 | |
| ACEI/ARB | 1.145 (0.799-1.641) | 0.462 | |
| β-Blocker | 1.367 (0.902-2.070) | 0.141 | |
| Antiplatelet medication | 1.223 (0.303-4.975) | 0.777 | |
| Stains medication | 1.199 (0.444-3.236) | 0.720 | |
| Anti-diabetic agents | 1.231 (0.897-1.689) | 0.198 | |
| Metformin | 1.607 (1.140-2.265) | **0.007** | |
| Alpha-glucosidase inhibitor | 0.955 (0.665-1.491) | 0.982 | |
| Sulfonylurea | 2.204 (1.314-3.699) | **0.003** | |
| SGLT2i | 1.281 (0.891-1.842) | 0.182 | |
| Dipeptidyl peptidase-4 inhibitor | 1.476 (0.547-3.983) | 0.442 | |
| Insulin | 0.767 (0.415-1.417) | 0.397 | |
| GLP-1 receptor agonist | 0.927 (0.296-2.906) | 0.896 | |
| TC (mmol/L) | 0.958 (0.806-1.139) | 0.625 | |
| HDL-C (mmol/L) | 0.910 (0.548-1.511) | 0.716 | |
| LDL-C (mmol/L) | 0.939 (0.760-1.161) | 0.563 | |
| HbA1c (%) | 1.033 (0.921-1.159) | 0.580 | |
| UA (umol/L) | 1.003 (1.001-1.004) | **0.001** | |
| eGFR (mL/min/1.73 m^2^) | 0.982 (0.973-0.990) | **<0.001** | |
| TyG index, per 1-unit | 1.363 (1.067-1.741) | **0.013** | |

HR, hazard ratio; CI, confidence interval; BMI, body mass index; SBP, systolic blood pressure; DBP, diastolic blood pressure; CVDs, cardiovascular diseases; PAD, peripheral artery disease; CKD, chronic kidney disease; ACEI, angiotensin-converting enzyme inhibitors; ARB, angiotensin II receptor blockers; SGLT2i, sodium-glucose cotransporter 2 inhibitor; GLP-1, Glucagon-like peptide-1; TC, total cholesterol; HDL-C, high density lipoprotein cholesterol; LDL-C, low density lipoprotein cholesterol; FBG, fasting blood glucose; HbA1c, glycated hemoglobin A1c; UA, uric acid; eGFR, estimated glomerular filtration rate. TyG index, triglyceride-glucose index.

^a^ Defined as eGFR<60 ml/min/1.73 m^2^ on the basis of The KDIGO CKD Clinical Guideline. Statistical significance was defined as *P*<0.05.

**Table S2. Sensitivity analysis for the association between the TyG index and outcomes.**

| **Outcomes** | **HR (95% CI)** | |  | |
| --- | --- | --- | --- | --- |
|  | **Analysis 1** | | **Analysis 2** | |
| TyG index as a continuous variable | |  | |  |
| Per 1 Unit increase |  | |  | |
| Primary endpoint events | 2.79(1.61-4.82)^***^ | | 1.77 (1.15-2.74)^*^ | |
| ASCVD events | 2.95(1.61-5.40)^***^ | | 1.72 (1.07-2.76)^*^ | |
| Per 1 SD increase |  | |  | |
| Primary endpoint events | 1.85(1.33-2.57)^***^ | | 1.41 (1.08-1.83)^*^ | |
| ASCVD events | 1.91(1.33-2.75)^***^ | | 1.38 (1.04-1.84)^*^ | |
| TyG index as a nominal variable |  | |  | |
| Primary endpoint events |  | |  | |
| T1 | 1 [Reference] | | 1 [Reference] | |
| T2 | 1.52(0.84-2.76) | | 1.24 (0.74-2.06) | |
| T3 | 2.92(1.51-5.62)^**^ | | 2.07 (1.20-3.56)^**^ | |
| P for trend | 0.006 | | 0.023 | |
| ASCVD events |  | |  | |
| T1 | 1 [Reference] | | 1 [Reference] | |
| T2 | 1.56(0.82-2.96) | | 1.34 (0.77-2.32) | |
| T3 | 3.30(1.63-6.69)^**^ | | 2.14 (1.18-3.87)^*^ | |
| P for trend | 0.004 | | 0.040 | |

**Analysis 1**: Excluding patients with a history of anti-diabetic agent usage.

**Analysis 2**: Excluding patients with a history of SGLT2i agent usage.

Multivariable Cox regression Model was adjusted for age, gender, current smoking, BMI, diabetes, established CVDs (including MI, stroke, HF), stains, antiplatelet drugs, fibrates drugs, SBP, DBP, UA, eGFR, TC, LDL-C in the multivariate model.

HR, hazard ratio; CI, confidence interval; BMI, body mass index; CVDs, cardiovascular diseases; MI, myocardial infarction; HF, heart failure; SBP, systolic blood pressure; DBP, diastolic blood pressure; UA, uric acid; eGFR, estimated glomerular filtration rate; TC, total cholesterol; LDL-C, low-density lipoprotein cholesterol. ^*^*P* < 0.05, ^**^*P* < 0.01, ^***^*P* < 0.001.

**Table S3. Multivariable Cox regression analysis for the association between the TyG index and outcomes.**

| **Outcomes** | | **HR (95% CI) ^a^** |
| --- | --- | --- |
| TyG index as a continuous variable |  |  |
| Per 1 Unit increase | |  |
| Primary endpoint events | | 1.51 (1.06-2.14)^*^ |
| ASCVD events | | 1.59 (1.08-2.33)^*^ |
| Per 1 SD increase | |  |
| Primary endpoint events | | 1.28 (1.04-1.58)^*^ |
| ASCVD events | | 1.32 (1.05-1.66)^*^ |
| TyG index as a nominal variable | |  |
| Primary endpoint events | |  |
| T1 | | 1 [Reference] |
| T2 | | 1.44 (0.91-2.28) |
| T3 | | 1.74 (1.07-2.85)^*^ |
| P for trend | | 0.018 |
| ASCVD events | |  |
| T1 | | 1 [Reference] |
| T2 | | 1.40 (0.85-2.32) |
| T3 | | 1.84 (1.08-3.13)^*^ |
| P for trend | | 0.014 |

**^a^** The control status of hypertension was included as a categorical variable and the continuous variables (SBP and DBP values) were excluded in the Multivariable Cox regression model 3. Model 3 was adjusted for age, gender, current smoking, BMI, diabetes, established CVDs (including MI, stroke, HF), stains, antiplatelet drugs, fibrates drugs, anti-diabetic agents, control status of hypertension, UA, eGFR, TC, LDL-C.

HR, hazard ratio; CI, confidence interval; BMI, body mass index; CVDs, cardiovascular diseases; MI, myocardial infarction; HF, heart failure; SBP, systolic blood pressure; DBP, diastolic blood pressure; UA, uric acid; eGFR, estimated glomerular filtration rate; TC, total cholesterol; LDL-C, low-density lipoprotein cholesterol. ^*^*P* < 0.05.
