# Supplementary material for: Triglyceride–glucose index as a marker of adverse cardiovascular prognosis in patients with coronary heart disease and hypertension
Source: Cardiovasc Diabetol. 2023 Jun 9;22:133. doi: 10.1186/s12933-023-01866-9 (PMC10257289; doi:10.1186/s12933-023-01866-9)

**Additional file 2**

**Additional Figure**

**Fig.S1. Kaplan–Meier curves for endpoint events according to quartiles of TyG index.**

Fig. S1 legend: A: Kaplan–Meier curves for primary endpoint events; B: Kaplan–Meier curves for ASCVD events.


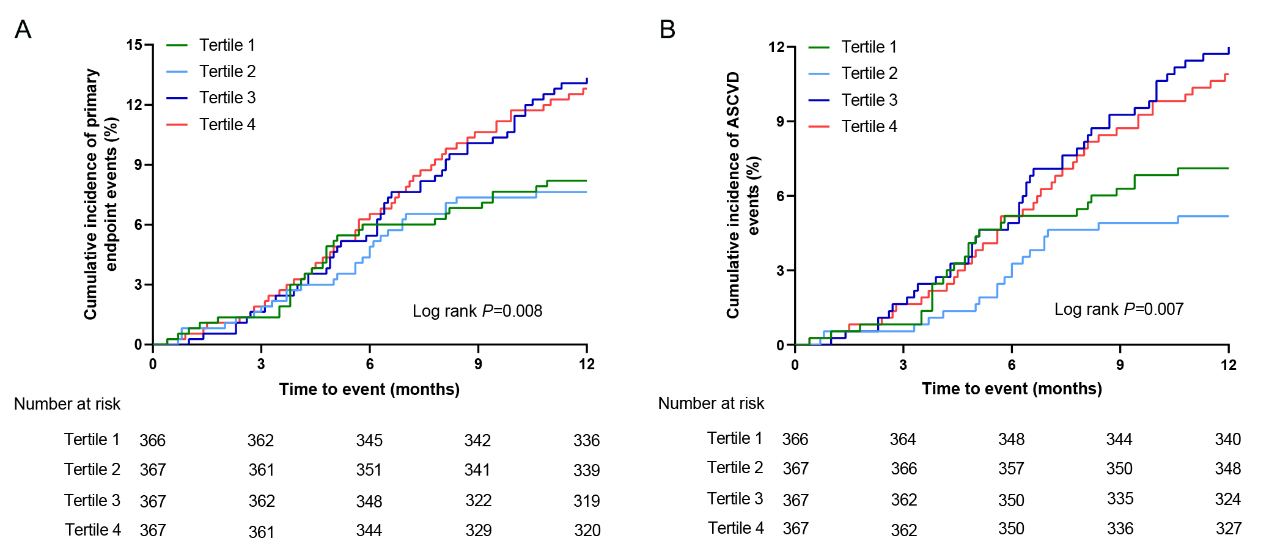

Supplement: Supplementary file 2 — Additional file 2: Figure S1. Kaplan–Meier curves for endpoint events according to quartiles of TyG index. A: Kaplan–Meier curves for primary endpoint events; B: Kaplan–Meier curves for ASCVD events. [file 12933_2023_1866_MOESM2_ESM.docx]
